# Extra Supplement—The Nursing Mirror

**Published:** 1889-02-02

**Authors:** 


*The Hospital*, FEBRUARY 2, 1889.

### The Nursing Mirror.

[*Extra Supplement.*

All Contributions for this Supplement should be addressed "The Nursing Mirror," care of The Editor, 38, Old Jewry, E.C.

### En Passant.

A BRAVE NURSE.—The Vicar of Westham, Hastings, records another deed of unostentatious bravery. Nurse Still, of the St. John's Community, while nursing a child with diphtheria who had undergone tracheotomy, sucked the tube, which became so clogged as to nearly cause suffocation.

ST. THOMAS'S HOSPITAL.—A pretty scene was witnessed in the Governors' Hall here on January 24th, when the annual concert was given to the nursing staff in South London. Besides the large number of nurses in uniform there were many visitors present, and nearly all the medical staff. An admirable concert was given, under the conductorship of Mr. Alfred J. Caldicott, and all enjoyed a very pleasant evening.

A CHRISTMAS TREE AT PERTH.—The lady nurses of the Perth Sick Nursing Society gave their annual Christmas tree to past and present patients on January 4th. The guests numbered 200, and were first treated to a substantial tea, and then to some speeches and songs. After that the serious work of the evening began, and the beautiful tree was stript of its presents, which were distributed amongst the company. Miss Rust presented the tree and many useful gifts. Mr. Pullar and the Rev. Mr. Manuel made the speeches.

DISTRICT NURSING AT ASHTON.—The work of the Ashton-under-Lyne Sick Nursing Association is growing rapidly, and the cry is now for a third nurse. In June last Miss Merritt (now matron at Penzance) was appointed maternity nurse to the Association, and did good work for two months She was followed by Nurse Summerfield, of Manchester, who is still working for the Association. At the annual meeting last week the Rev. Thomas Green made an amusing speech, in which he reminded his hearers that there were three Christian virtues, and that in exercising charity, they should not forget faith and hope: he advised them to secure the services of a third nurse, and trust to a rise in the receipts to meet the extra expense.

A NURSES' BALL.—This interesting event, which took place on the 18th ult. at Nottingham General Hospital, was again [a great success. All the nurses (about thirty), the matron, the resident staff with their friends, and most of the old residents were present. The ball was held in a large ward, known as the "Old Wing." Dancing commenced at half-past eight to the strains of an excellent band; and during the evening a programme consisting of twenty-four dances was got through. The nurses, who wore their pretty uniform, looked particularly well. It has been found that this ball, which takes place annually, does not in any way interfere with the discipline of the hospital, but, on the contrary, has done much towards promoting a good feeling throughout the house.

NOTES FROM AMERICA.—A Memorial Training School for Nurses has just been established at Pittsfield, Mass., by Mr. Henry W. Bishop, of Chicago, as an evidence of his appreciation of the value of the services rendered by trained nurses to his son during his fatal illness at Pittsfield, and also with a desire to aid in extending the widening field of usefulness of trained nurses.—The Training School for Male Nurses at Bellevue Hospital was formally opened on December 17th. The building has accommodation for 50 persons. Out of 113 applicants, 22 were selected. They will be given a two years' training similar to that of the female nurses. An order has been issued at Bellevue Hospital, New York, that the histories of all patients are to be kept secret from all save those who bear an order from the board. This is owing to the incursions of journalists and interviewers.

MASSAGE.—An excellent paper on this subject appears in *Work and Leisure* for January. The writer, Miss M. E. Thonger, deals entirely with her own experience, and in her own language explains as much as is explainable by description of the different methods of massage. The writer states that she seldom finds it possible to work without using either oil or violet powder, and that as a rule she uses filtered neat's-foot oil, scented with a little oil of cedar-wood. A typical case is described, where general massage is performed, beginning with the toes and working upwards till the whole body has been systematically gone over, finishing off with the hands and arms. The usual time for one treatment is an hour, and it is said that the artificial exercise to the patient during this hour is equal to a two-mile walk. Miss Thonger insists that massage is a work of head and heart as well as of hand, and should only be taken up by those who have a natural aptitude for dealing with sick folk.

A MEDICAL MISSION STORY.—In the *Christian* for January 11th there is a story which tells of some of the good wrought by the nurses of the Mildmay Mission Hospital, Bethnal Green. A man of about sixty, with his right hand crushed, is found in a cold, dirty, untidy garret. His left hand had been amputated (without chloroform) in '37, and one heel had been operated on, and left him crippled. After these experiences the poor man stoutly refused to go to a hospital to have his right hand attended to, and he did not welcome too cordially the services of the Mildmay nurses. However, they gradually grew in his favour, and he consented to be operated on, and then he steadily improved in health. He was chiefly touched by a gift of "baccy" from the minister. His ten-years-old daughter used to light his pipe and put it between his teeth before she went to school, and there it would be when she returned again. He has gone back to his work now, and is obliged to hire a boy to pull the barrow. His hand, though much improved, is still stiff; there is just sufficient movement in the fingers to enable him to handle money, but he can't fasten a single button himself. This difficulty he has overcome by ingenious contrivances, which are really quite a study. He is a teetotaller, and attends church now, though formerly a scoffer at religion and a drinker. The whole case affords an illustration of the power of consecrated lives.

THE BRITISH NURSES' PLANS.—A correspondent is much exercised on this question, and so far as we gather, his opinions are these: "The British Nurses' Association was started avowedly to procure legal registration of nurses, which it has not of course procured; yet it is now announcing its intention of starting various other schemes, including a benevolent fund, for which there is absolutely no room, all that ground being covered by the Pension Fund, which is legally constituted for that purpose, amongst others under Acts of Parliament. He takes it for granted Miss Wood has not got £20,000 to pay down to secure the registration of her fund; would it not therefore be more businesslike to finish one plan before starting half-a-dozen others? When the secretary has succeeded in registering all nurses, whether they like it or not, then he will believe she can found a fund which can also be legally registered. In the memorandum of the objects of the Pension Fund, our correspondent says, provision is made for life assurance, pensions, and insurance against illness or accident, and also for a special relief fund for sad cases which may call forth the generosity of the lady patronesses of the society. Too many nurses are utterly ignorant that in joining the Pension Fund they are becoming eligible for all these advantages. In any case a benevolent fund founded on the wretched voting system would be a disgrace to these days of advanced philanthropy. It results in much wasted labour, and keeps the poor petitioners on tenterhooks for months, often dooming them at last to bitter disappointment." We are sure, if our correspondent would call on Miss Wood to explain his views, he would meet with a warm reception.

### Notes from Australia.

(*By Our Correspondent.*)

MELBOURNE, DECEMBER 1ST, 1888.

THE proper training and treating of nurses is coming daily to the fore in Australia, and soon we hope our system will equal that prevalent in London hospitals. A lecture on nursing was lately delivered by Dr. R. Schlesinger to the pupils in training and the nursing staff of the Alfred Hospital. "Surgical Nursing" formed the subject of the lecture, and an intimation was given that the next lecture by Dr. Schlesinger would be on the subject of "Surgical Dressing." Twenty of the staff and six lady visitors attended the lecture.

At the late meeting of the committee of the Women's Hospital, a communication was received from a number of pupil nurses at the institution, petitioning to have one half-day off duty in every week, and every third evening off after six p.m. Under existing arrangements they were on duty for fourteen hours daily—*i.e.*, from six a.m. till eight p.m.—after which they were too much fatigued to get the exercise and change which were indispensable for the fulfilment of their duties. The demand was a just one, for now the nurses have to ask leave whenever they would go out, and though leave is never refused, still the right to proper periods of recreation should be theirs, without having to ask it as a favour. It is strange that these questions should come forward so often in Australia, where there are women on the boards of nearly all the charities. Mrs. Goe is president of the Women's Hospital, and might have had more sympathy with the needs of her sex. In England, where men alone form hospital committees, I believe the nurses are treated more considerately. Mrs. M'Kay, the matron of Melbourne Hospital, is away on leave, as she has had very bad health lately. Mr. Willder has raised the £10,000 to clear the debt on Melbourne Hospital. Mr. J. S. Butters has addressed a circular note to the board of management of all the charitable institutions in the colony outside Melbourne, asking to be furnished with a statement of the indebtedness of each, and he has now received replies which show that the total sum needed to clear off the whole of the liabilities on these institutions is £17,123 4s. 6d. Mr. Butters intends taking measures during next week to solicit the aid of all willing to assist in wiping off this large debt. Certainly in their prosperity Australians do not forget to be generous, and they readily give the money they easily obtain.

The following public lectures have been lately delivered by the Australian Health Society: October 29th, "Causes of Blindness," by Dr. J. W. Barrett; November 5th, "Heart and Brain," by Dr. Usher, F.R.G.S.; November 12, "Brain Growth and Education," by Dr. Nylulasy; November 19th, "Microbes and Disease: A Result of Fifty Years' Investigation," by Mr. A. N. Pearson, the Government agricultural chemist; November 26th, "Water Supply of Towns," by Professor Orme Masson. Mr. Pearson's lecture on "Microbes and Disease" was most eloquent, and drew a large attendance. The lecturer began by referring to the diseases, particularly that of leprosy, which prevailed among the ancient Egyptians, and then entered upon the wide subject of fermentation. After making an allusion to Schwann's discovery in reference to the great similarity which existed between fermentation and putrefaction, the lecturer described the low forms of life which existed in mushrooms and cheese, and extolled the successful experiments which M. Pasteur had made in regard to microbes and their connexion with hydrophobia, chicken cholera, etc. A paper on the "Hereditary Effects of Alcohol," read by Miss Jessie Fowler at the Women's Christian Temperance Conference, has also been much discussed.

The case of Dr. Laura Morgan is to the fore again. In 1885 she applied to the Medical Board of Victoria to be registered as a medical practitioner. She sent in documents, including a diploma from the Women's Medical College, New York Infirmary, certificates from the New York Homœpathic Medical College, and certificates from various consuls. These documents were held not to prove conclusively that Dr. Laura Morgan had passed three years at her medical studies, and she was requested to provide further proofs. In January, 1888, further evidence was tendered to the board, but they still remained unfavourable. Miss Morgan has now put a plate on her door, and is practising until proceedings shall be taken against her. This does not sound hopeful for lady doctors who may think of visiting us from England.

Three years ago Lady M'Culloch, wife of Sir James M'Culloch, on her departure from Victoria, relinquished to a committee of ladies the management of the Convalescent Home for Women, succesfully established and carried on at her sole expense for a number of years. The requirements of the Home have greatly increased, and the energetic committee have obtained the funds to erect a new and larger Home. On November 21st Lady Loch laid the foundation-stone of the new structure, which is to cost £13,000. The plans show a general design, cosy and home-like, and the invalids will have the benefit of all the latest improvements.

The December number of the *Australian Medical Journal*, out to-day, gives particulars of the Intercolonial Medical Congress of Australasia, to be held in Melbourne in January. Baron Sir Ferdinand von Mueller will deliver the address in Pharmacology. Dr. Stirling, of Adelaide, will deliver the address in Surgery, and Dr. Batchelor, of Dunedin, that in Obstetrics and Diseases of Women. Dr. M'Laurin, the medical adviser of the Government of New South Wales, will deliver the address in Hygiene, Forensic, and State Medicine. This will be followed by the address in Pathology, given by Dr. Bancroft, of Brisbane. The general meetings to discuss the fevers of Australasia and hydatid disease naturally attract attention. But, in addition, the sectional proceedings will include important papers on such subjects as phthisis and diphtheria; and the sections of state medicine and psychological medicine will be almost as attractive to laymen as to members of the congress. In the former section, papers will be submitted on the present state of sanitation in the different colonies; and in the latter, the subjects dealt with will include the prevalence of insanity in Australasia, its curability, the establishment of receiving houses, the classification and housing of the insane, the training of asylum attendants and nurses, and other matters which are now engaging attention in the various legislatures.

Dr. Matthews, of Branxholme, gives the following account of the outbreak of diphtheria at that place: On November 12th he was called to see a boy of Mr. Best's, aged four years, at eight a.m. He was suffering from advanced diphtheria laryngitis, and died six hours after. The same evening, at six o'clock, he saw another boy in the same family, aged nine, who had been complaining of his throat, but the patient did not speak to him of it when he called in the morning. He was suffering from advanced laryngeal diphtheria, and died at twenty minutes to ten the next morning. A boy aged seven was found to be suffering from tonsillar diphtheria, a patch of membrane the size of the thumbnail on left tonsil. The next morning right tonsil affected also. November 13th—Boy aged nine found to be suffering from tonsillar diphtheria. November 12th—Miss Best, aged 16, small patches on tonsils when seen at six p.m. Result—two deaths and three recoveries. The two former were past treatment when first seen, the breaking being very much implicated. Cause—"A murderous state of insanitation." There can be no doubt that sewage disposal and general sanitation is the burning question here just now.

### A Christmas Holiday.

*(Continued from page lxviii.)*

PART III.—THE FIRST COTTAGE HOSPITAL.

WE were very tired and stiff next morning, and groaned very much during the dressing process, but by the time we had had breakfast we had recovered our spirits, and the stiffness was wearing off. Guildford was very quiet this Sunday morning, and, resisting the entreaties of our friends, we determined to start off before the church-goers were about. Horsham was only nineteen miles, and we hoped to be there in time to attend evening service. We strolled slowly out of the town, having received orders to go via Bramley and Cranleigh. The first signpost we came to pointed one way for Cranleigh and the other for Bramley, and as we understood we were to go to both places we were somewhat perplexed. We finally took the Bramley road, and reached that little village just as the inhabitants were going to church. In a small shop here we looked with longing eyes at some old brass ladles and worm-eaten rushlight-holders, Had it not been Sunday we would gladly have done a deal at that old shop. Presently we reached Cranleigh, but as it was not yet one we determined not to stop for lunch. "Oh, look, what a dear little village hospital! And built in 1859, too! None of your modern Jubilee constructions," cried Nurse Sadler.

Yes, there in the very centre of the village was a gabled cottage with diamond-paned windows, and bearing the inscription, "Village Hospital, 1859." We both hesitated; but if we were ever to get to Brighton it was necessary to march firmly onwards; so I caught hold of Nurse Sadler's arm and dragged her away. We had got about a mile beyond Cranleigh when it began to rain—a slow, steady rain which looked like lasting. We took shelter under a cart-shed beside the road, and waited awhile, and considered what was our best plan. As we only carried a small hand-bag each, and had no gowns to change, we did not like to face walking nine miles to Horsham in a downpour, and yet to turn back is always a melancholy proceeding. Some cocks and hens shared the cart-shed with us, and Nurse Sadler suggested we should stay where we were; kill and cook a fowl for food, and sleep in the cart. But, though on tramp, I had no desire to be also a vagrant; so sorrowfully we returned that mile to Cranleigh, and put up at a comfortable little inn.

"Now we can hear all about the hospital," said Nurse Sadler, and forthwith she questioned the whole household, and gained the following information: The little hospital, originally founded by the village surgeon, Mr. Albert Napper, F.R.C.S., whose memory is treasured by the villagers, was much appreciated by those for whom it was designed and supported. It was free to every case of accident or emergency, and seemed to have few rules and many comforts. "It was almost the same as being at home," one man said. The rector of the parish, the Ven. Archdeacon Sapte, is the main support and manager of the small hospital, and the expenses for the year, during which some thirty patients are treated, usually amount to about £150. Cranleigh was the first village hospital erected in England, and can look down with scorn on all the modern cottage hospitals. We had some thought of applying to be taken on at Cranleigh Hospital, and ending our days in that quiet, pretty little place, but we were informed there was only one nurse and a "woman what scrubbed;" and as we would neither consent to be the "woman what scrubbed," we abandoned the plan. We went to church that evening, and were disappointed not to hear the rector preach.

Monday morning dawned dull and wet again, and, after a long consultation, we were forced to forego the rest of our walking tour, and to be content to make the best of our way to Brighton by train. When we arrived at that place about noon we found a glorious sea breeze blowing, which had driven all rain in-land, and treated us to salt spray instead. We went straight down on to the beach as close as we could get to the great rolling waves, and we drank in the invigorating sea-air with the keenest delight. Hunger at last drove us to seek our friends, and we reluctantly left the beach.

*(To be continued.)*

### Modern Prophets on Truthfulness.

A CORRESPONDENT writes:—Certainly neither doctors nor others are agreed at present on the question of veracity. In the January number of the *Nineteenth Century* Oscar Wilde wrote an article on the "Decay of Lying," in which he deeply regretted the loss of an art which lends beauty and pleasure to life. Then, on January 18th, Mr. Brudenell Carter expounded the theory that lack of truthfulness is an awful calamity, against which we must all strive. Again, in the current number of the *Universal Review*, Dr. Lauder Brunton gives us an article on "Truth and Delusion," which practically upholds that one man's lie is another man's truth, and that both are more or less a delusion. Amongst such conflicting opinions where are we to seek counsel? Are we to follow the advice of Oscar Wilde and Miss Mollett, and try and adorn the bare fabrics of fact with a little fiction, which will round off the harsh corners of every-day life, and hide a few of the skeletons that not only repose in cupboards in these days, but which stand naked at every corner? Or are we to listen to the grand denunciation of Brudenell Carter, and forsake every vestige of fiction, and stand fast on the bare rock of precise truth; confining our conversation to "Yea, yea; nay, nay;" lest we fall from perfect veracity and are counted amongst the liars? Or are we to listen to Lauder Brunton's mild philosophy, and talk about opinions and delusions, and acknowledge that truth has two sides? From Plato downwards we have had endless philosophers ready to argue on the knotty point of veracity, but whether much practical good has come from these dissertations is another matter. Looking at the question from a commonplace view, we think many nurses will agree with us that in dealing with the serious business of life no standard of truthfulness can be too strict; but that in dealing with the pleasures of life, yea, yea, and nay, nay, is somewhat monotonous, not to say dull. A world void of imagination would never have discovered many of the truths which now comfort and sustain us; and the nurse who has not a poetic ideal before her, will scarcely struggle above the ordinary level. Really we feel inclined to beg Mr. Brudenell Carter to "leave us our lies!" When the work of the day is over, and we have devoted long hours to scientific truthfulness of word and deed, how refreshing and cheering is a dose of fiction! Though it come as conversation, play, or novel, it is equally welcome, and with true womanly weakness we love the "embroidery" of which Mark Pattison accused Sister Dora. Facing the stern facts of life, as a nurse must throughout her time on duty, a little relaxation to indulge in beautiful, if vain, imaginings should surely be hers. Let us then choose of the modern prophets Lauder Brunton for hours of leisure, for he walks in the middle path, whilst we follow Mr. Brudenell Carter in every serious matter affecting our work and daily life.

[*Correspondence on all subjects is invited. No communications can be entertained if the name and address of the correspondent is not given, or unless one side of the paper only be written on.*]

### OAT FLIGHT.

T. A. writes :—Allow me to tell you that "oat flight" is the chaff produced in the process of threshing oats. It is sifted and used for filling beds, considered by some authorities as very cleanly for that purpose, as after a death or what not the oat flight can be destroyed, and the tick cleansed. Three years ago, all the patients in the Borough Hospital, Leicester, were nursed on such beds, with bolsters and pillows *en suite*; no doubt it is the same now—that I cannot say. You can imagine how difficult it is to get a nicely-shaped bed, and the dust produced whilst making them is most unpleasant. Poor people in rural districts avail themselves of oat flight beds, as of course they are very inexpensive. Owing to the wet harvest season, the flight is very dirty this year, and I conclude that is why the E. and C. G. H. have to advertise their need of it.

### COMBINATION OF PRIVATE NURSES.

Nurse Grant writes: For the past two years I have been engaged in private nursing. How much I have wished I had stayed in the infirmary, comfortless though it was, would be hard to say, for then I could always think the poor patients were grateful to me for what I did for them, but now I feel nothing of the kind. The institution here belongs entirely (without a committee) to two nurses. The one aim is clearly shown in the treatment we receive by being sent off from one case to another (sometimes having been up the previous night) as being nought higher than money. Out of all we earn we only get £20 a-year. The nurses are hardly ever in a day, there seems to have been such an amount of illness here. I think most of the nurses are very dissatisfied, and if you favour me with an account of how we can combine for our own protection I will do my best to forward the scheme here. Would it be well to call a large meeting of private nurses?

Nurse M. B. writes: I want to let you know how thankful I feel to see in the last issue of The Hospital how you are kindly interesting yourself in a combination of private nurses for their own protection. If, in addition to all the other good you have done for trained nurses, you could help them to get work for which they (not others) would be paid, we should all be grateful and glad to help you.

### REST AND EXERCISE.

F. W. T. writes:—I am glad to see in The Hospital that the subject of rest and exercise is taken up by a nurse. I hope it may be taken up by many, and meet the eyes of some ladies employing us as nurses. Is it to be wondered at that nurses break down when one comes to inquire how many hours they spend in the sick-room? So may the subject be considered, for our salary is but small, and our health is our only capital; when that fails it means lesser earnings, sore exhaustion, and the nurse who has given the best years of her life to tending the sick frequently finds herself thrown upon the world's sympathy with no other resource save a miserable pittance from the parish with which to eke out her existence. Perhaps some may say there is the Pension Fund—far from our reach with our miserable salary, and if one did join, what use is it to have to wait till we are sixty till we get anything, for what hospital or institution will employ till then, or how fit shall we be for our anxious post? [If F. W. T. will not do something, however small, for herself, how can she ask help of others?—Ed. *T. H.*]

### NOT ONLY WOMEN, BUT ANGELS.

An Old Westminster Nurse writes: Seeing the letters in last week's Hospital, I felt I must send the following: My cousin, John M., had a lumbar abscess, and the doctors advised removal to St. Bartholomew's Hospital. He was unwilling to go from his kind attentive wife, but was at last prevailed upon to do so. I visited him there, and he spoke of the nurses' kindness and tender consideration of his feelings while performing the disagreeable duties of nursing; and one day he said, suddenly, "Ruth! who are these nurses? They are not only women, but angels sent from God." His last hours were spent under their care.

### A CONTENTED PROBATIONER.

A Contented Pro writes: I should like to say a word on the "Contented Pro's" side, and that is, that the three years I spent as such were the happiest days of my life. When on duty we worked very hard, but we were happy and cheerful together, so forgot the hardness of the work. We were never allowed to talk what our matron called "shop" out of our wards, to make us forget our work for a time. There was some little pleasure for the evening, such as an invite to the sister's room for music and coffee, or to the matron's room for a lecture, which we enjoyed most. Then our committee gentlemen were very kind, sending us to the theatre or a concert. Of course we had the rough side to contend with, but there are some people who think, in becoming a nurse, there is nothing to do in an hospital, only shaking up a patient's pillow, or giving him a drink.

### Appointments.

Gateshead Children's Hospital.—Miss E. R. Miles, of the General Hospital, Swansea, has been appointed matron at Gateshead. Miss Miles has had much experience with children, and has some excellent testimonials. She trained at the Evelina, and was afterwards senior head nurse at Victoria Hospital, Burnley. From there she went to the Alexandra Hospital at Brighton, where she had charge of the boys' wards. We congratulate Miss Miles on her new appointment.

Canterbury Nurses' Institute.—Miss C. N. Harbord has been appointed matron of the Kent and Canterbury Nurses' Institute. Miss Harbord trained in the Victoria Hospital for Children, and one year in Guy's Hospital; subsequently she had experience of nursing in private cases, and at the Cumberland Infirmary, Carlisle, where she also acquired an insight into house management. Since September last she has been engaged as one of the nurses in the Kent and Canterbury Institute. The institute now has a staff of nine nurses, one district nurse, and four probationers. The demand for the nurses exceeds the supply.

### Presentations.

On January 24th Miss E. Reynolds, for fifteen years Lady Superintendent of the Adelaide Hospital, Dublin, was presented with an address, a clock, and a purse of sovereigns. Miss Reynolds has made many friends during her nursing career, but the remarkable influence she has ever exercised over the medical students of the hospital is the most unique trait in her character. Nearly all the students attended on Friday last, and gave three cheers for Miss Reynolds when she finished the touching speech in which she acknowledged the presentation.

### Vacancies.

The following vacancies are announced :—

**Matron.**

Northumberland County Asylum, £70.

**Lady Superintendents.**

Kensington District Nursing Association.
Convalescent Home, Matlock; £40.

**Nurse Matron.**

Southend Victoria Hospital; £30.

**Nurses.**

Nursing Institution, 96, 97, 98*a*, Wimpole Street, London, W., Mr. Wilson has several vacancies for thoroughly-trained Nurses.

South Shields Union, £25.

Borough Hospital, Sheffield; £22.

Herne Bay District School; £25.

Wonford House Asylum, Exeter.

Truro Nurses' Home; £25.

Woolton Convalescent Home, near Liverpool.

Bolton Infirmary.

Tain Poorhouse; £25

St. Heliers Home; £25.

Norwich Nurses' Home.

National Hospital, Queen's Square £20.

Shoreditch Infirmary; £20.

Bangor Nursing Institute; £20.

Southport Nurses' Home.

Brockley Nursing Home.

County Institution, Worcester.

Southport Trained Nurses Home; several.

Glasgow Sick Poor Association.

Firs Home, Bournemouth; £20.

**District Nurse.**

Bedford Nursing Institute.

**Midwives.**

Glasgow Sick Poor Association.

**Probationers.**

Leeds Nurses' Institution; £14.
Great Yarmouth Hospital.
Brighton Borough Fever Hospital; £16.
Seamen's Hospital, Greenwich.
Tewkesbury Hospital.
Coldstream Cottage Hospital.

**Wardmaids.**

Brighton Borough Fever Hospital; £16.
Halifax Infirmary.

### Notes and Queries.

**Queries.**

(105) *The Dispensary Doctor.*—By whom is this song published?—*Ignoramus.*

**Answers.**

(100) *Home Wanted.*—Get Low's "Handbook of Charities," it will be sure to tell of such a home.

(102) *Training in Midwifery.* — Try Brownlow Hill, Liverpool, or Female Hospital, Woolwich.

(104) *Starting a Crèche.* — Mrs. Hilton, of 16, Stepney Causeway, London, E., has started and worked a splendid crèche. Doubtless she would kindly give particulars.

*Nurses and Stewardesses.*—Birmingham, Bristol, New Cross, Sheffield, and other correspondents, are informed that their names are entered on the register of nurses ready to act as stewardesses.

*To Correspondents.*—Several letters are unavoidably held over till next week.

